# Hyperactive RAS/PI3-K/MAPK Signaling Cascade in Migration and Adhesion of *Nf1* Haploinsufficient Mesenchymal Stem/Progenitor Cells

**DOI:** 10.3390/ijms160612345

**Published:** 2015-06-01

**Authors:** Yuan Zhou, Yongzheng He, Richa Sharma, Wen Xing, Selina A. Estwick, Xiaohua Wu, Steven D. Rhodes, Mingjiang Xu, Feng-Chun Yang

**Affiliations:** 1 State Key Laboratory of Experimental Hematology, Institute of Hematology and Blood Diseases Hospital, Chinese Academy of Medical Sciences and Peking Union Medical College, Tianjin 300020, China; E-Mail: xw2318@aliyun.com; 2 Department of Pediatrics, Indiana University School of Medicine, Indianapolis, IN 46202, USA; E-Mails: yonhe@iu.edu (Y.H.); selinaorlando@yahoo.com (S.A.E.); wu_xiaohua@lilly.com (X.W.); mx2@iupui.edu (M.X.); fyang@iu.edu (F.-C.Y.); 3 Herman B Wells Center for Pediatric Research, Indiana University School of Medicine, Indianapolis, IN 46202, USA; E-Mails: ricsharm@iupui.edu (R.S.); sdrhodes@iupui.edu (S.D.R.)

**Keywords:** neurofibromatosis 1, neurofibroma, oncogene protein p21 (ras), mesenchymal stem/progenitor cells

## Abstract

Neurofibromatosis type 1 (NF1) is an autosomal dominant disease caused by mutations in the *NF1* tumor suppressor gene, which affect approximately 1 out of 3000 individuals. Patients with NF1 suffer from a range of malignant and nonmalignant manifestations such as plexiform neurofibromas and skeletal abnormalities. We previously demonstrated that *Nf1* haploinsufficiency in mesenchymal stem/progenitor cells (MSPCs) results in impaired osteoblastic differentiation, which may be associated with the skeletal manifestations in NF1 patients. Here we sought to further ascertain the role of *Nf1* in modulating the migration and adhesion of MSPCs of the *Nf1 haploinsufficient* (*Nf1^+/−^*) mice. *Nf1^+/−^* MSPCs demonstrated increased nuclear-cytoplasmic ratio, increased migration, and increased actin polymerization as compared to wild-type (WT) MSPCs. Additionally, *Nf1^+/−^* MSPCs were noted to have significantly enhanced cell adhesion to fibronectin with selective affinity for CH271 with an overexpression of its complimentary receptor, CD49e. *Nf1^+/−^* MSPCs also showed hyperactivation of phosphoinositide 3-kinase (PI3-K) and mitogen activated protein kinase (MAPK) signaling pathways when compared to WT MSPCs, which were both significantly reduced in the presence of their pharmacologic inhibitors, LY294002 and PD0325901, respectively. Collectively, our study suggests that both PI3-K and MAPK signaling pathways play a significant role in enhanced migration and adhesion of *Nf1* haploinsufficient MSPCs.

## 1. Introduction

Neurofibromatosis type 1 (NF1), also known as Von Recklinghausen’s disease, is an autosomal dominant disorder with an incidence of 1 in 3000 live births [1]. NF1 is one of the most common genetic disorders with a predilection toward neoplasms, which include astrocytomas, pheochromocytomas, myeloid leukemia, and the pathognomonic cutaneous and plexiform neurofibromas [2]. In addition to these neoplastic conditions, NF1 patients variably experience skeletal deformations, hyperpigmentation of the skin (café-au-lait macules), benign lesions of the iris (Lisch nodules), and intellectual deficits [3,4].

NF1 occurs as a result of mutations in the *NF1* tumor suppressor gene located on chromosome 17p11.2, which encodes a p21ras (Ras) guanosine triphosphatase (GTPase)-activating protein (GAP) called neurofibromin. The neurofibromin GAP domain controls the conversion of Ras-GTP to its inactive GDP-bound state, thereby negatively regulating the activity of downstream signaling pathways, including the mitogen activated protein kinase (MAPK) and phosphoinositide 3-kinase (PI3-K) pathways. Loss of one or both alleles of *NF1* leads to aberrant Ras-dependent cellular functions including proliferation, differentiation, migration, and survival, in multiple cell lineages [5,6].

Mesenchymal stem/progenitor cells (MSPCs) was first isolated from bone marrow by Friedenstein in 1970 [7], follow-up studies demonstrated that they effectively support the hematopoietic stem/progenitor cell (HSPC) functions through expression of adhesion surface molecules, extracellular matrix, and cytokine production within the hematopoietic microenvironment, termed as “niche” [8,9,10,11]. MSPCs are identified as being positive for CD105, CD73, CD90, and negative for CD45, CD34 and CD117 [12] and account for 0.01%–0.0001% of all nucleated cells in the bone marrow [13]. MSPCs also retain the capacity for self-renewal and differentiation into many non-hematopoietic mesodermal tissues such as osteoblasts, adipocytes, and chondroblasts *in vitro* [7,14,15] and exhibit the potential to generate complete bone/bone marrow organs *in vivo* [8]. Furthermore, studies have shown that MSPCs produce trophic factors that promote their migration resulting in enhanced tissue repair, thereby providing therapeutic benefit in inflammatory disease processes and sites of injury [16,17]. Skeletal abnormalities, including osteoporosis/osteopenia, osteomalacia, shortness of stature, and macrocephaly are among the common non-malignant complications in patients with NF1, and some of these bone manifestations can result in significant morbidity. Recent studies indicated that the osseous manifestations in NF1 may due to the impaired maintenance of bone structure and abnormal development of the skeletal system [18,19,20]. Given that MSPCs are progenitors of osteoblasts, functional defects of MSPCs may be closely relevant to skeletal development.

Our previous studies have shown that heterozygous loss of *Nf1* (*Nf1^+/−^*) in MSPCs led to increased proliferation, hyper activation of p21-Ras and impaired MSPC differentiation into osteoblasts [21]. We have also demonstrated that haploinsufficiency of *Nf1* led to hyper activation of the Ras/PI3-K/MAPK signaling axis in Schwann cells, osteoclasts, and mast cells [22,23]. Till now, the molecular mechanisms underlying the gain-in-migration of NF1 MSPCs remains poorly understood and yet to be elucidated. We hypothesized that *Nf1* heterozygosity may also lead to alteration of MSPC cellular functions including migration and adhesion via p21-Ras mediated hyperactivation of PI3-K or MAPK effector pathways. In the present study, we utilize MSPCs derived from bone marrow of wild-type (WT) and *Nf1^+/−^* mice to investigate whether *Nf1* heterozygosity affects MSPC migration and adhesion capabilities.

## 2. Results

### 2.1. Nf1^+/−^ MSPCs Have Increased Nuclear-to-Cytoplasmic Ratio

**Figure 1:**
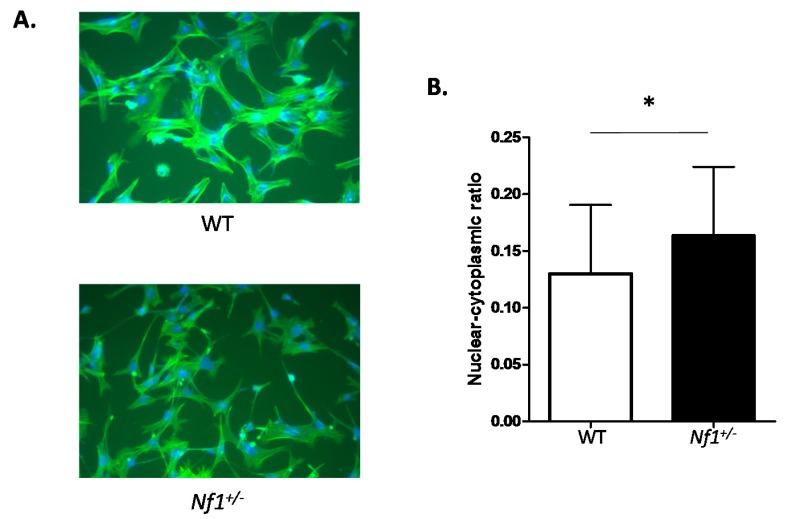
Morphological differences between wild-type (WT) and *Nf1 haploinsufficient* (*Nf1^+/−^*) mesenchymal stem/progenitor cells (MSPCs). (**A**) Morphology of WT and *Nf1^+/−^* MSPCs imaged under 200× amplification by phase contrast microscopy. Cells were stained with 400 nM fluorescein isothiocyanate(FITC)-phalloidin and DAPI; (**B**) A quantitative comparison of nuclear-cytoplasmic ratio between WT and *Nf1^+/−^* MSPCs based on the average ratio of nuclear area/cytoplasm area in 50 cells/field from five different fields. Data are represented as mean ± SD from three batches of MSPCs isolated from individual mice (* *p* < 0.05 for *Nf1^+/−^* *vs.* WT MSPCs).

*Nf1^+/^*^−^MSPCs were noted to have elongated, spindle shaped cytoplasm in comparison to the branched cytoplasm observed in WT MSPCs (Figure 1A). Quantification of this morphological change revealed an increased nuclear-to-cytoplasmic ratio in *Nf1^+/−^* MSPCs compared to WT controls (Figure 1B). These findings indicated involvement of neurofibromin in regulating MSPC morphology.

### 2.2. Nf1^+/−^ MSPCs Have Increased Migratory Capacity

**Figure 2:**
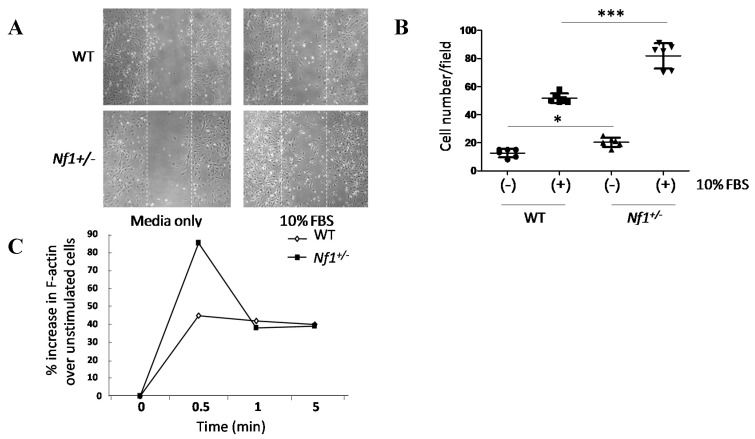
Migration and actin polymerization were significantly enhanced in *Nf1^+/−^* MSPCs. (**A**) Wound healing assays were performed by incubating WT and *Nf1^+/−^* MSPCs in 10 µg/mL of mitomycin C for one hour, after which a linear wound (marked by the white dotted lines) was created as shown. Wound healing was allowed to proceed in fresh media for 24 h, (original magnification ×200); (**B**) The number of cells migrating into the wound field were quantified, revealing an increased migration in *Nf1^+/−^* MSPCs compared with WT MSPCs (*F* = 75.76, Df = 1, *** *p* < 0.001; *** *p* < 0.001 for *Nf1^+/−^* MSPCs *vs.* WT MSPCs in the presence of 10% FBS, * *p* < 0.05 for untreated *Nf1^+/−^* MSPCs *vs.* untreated WT MSPCs). Data are represented as mean ± SD from duplicate wells from three independent experiments, each experiment was performed with different MSPCs culture isolated from individual mice; (**C**) Actin polymerization was measured following 2 h starvation and subsequent treatment with 10% FBS for different time periods. Flow cytometry analysis was performed following 400 nM FITC-phalloidin staining. An increased F-actin content was observed in *Nf1^+/−^* MSPCs comparison to WT MSPCs. A representative result of one of three independent experiments is shown; each experiment was performed with different MSPCs culture isolated from individual mice.

Wound healing assays was performed to assess migration of MSPCs. *Nf1^+/−^* MSPCs were noted to occupy an increased proportion of the wound space as compared to WT MSPCs 24 h after wound formation and continued culture in medium containing 10% fetal bovine serum (FBS) (Figure 2A). Quantification of the wound healing assay revealed a significant increase in the number of migrating *Nf1^+/−^* MSPCs (*F* = 75.76, Df = 1, *p* < 0.001; Figure 2B). Actin polymerization was further assessed to determine a molecular basis for the increased migration noted in *Nf1^+/−^* MSPCs. *Nf1^+/−^* MSPCs, as compared to WT controls, demonstrated significantly increased F-actin (polymerized actin) content after FBS stimulation at 30 s (Figure 2C, Figure S1). Taken together, these results suggested an increase in the migratory capacity of *Nf1^+/−^*MSPCs secondary to enhanced F-actin polymerization.

### 2.3. Nf1 Haploinsufficiency Enhances Cellular Affinity to CH271

**Figure 3:**
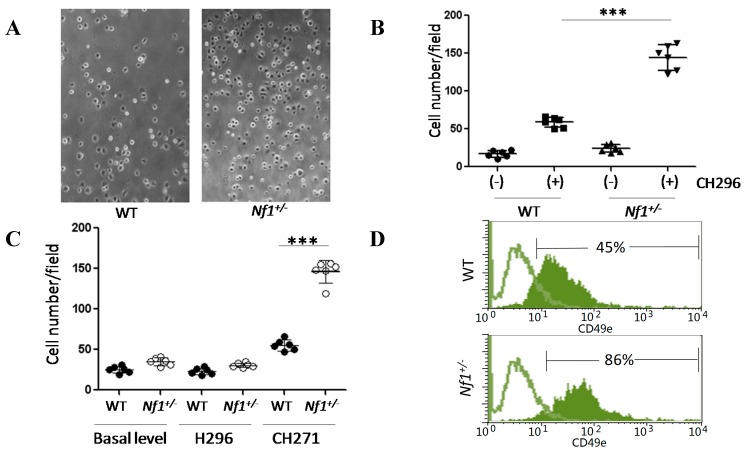
Enhancement of cellular adhesion in *Nf1*^+/−^ MSPCs. (**A**) MSPCs were plated into wells pre-coated with either 8 µg/mL CH296 (recombinant fibronectin fragment) or 0.1% bovine serum albumin (BSA). Following a 30 min incubation period at 37 °C, the plates were washed and adherent cells were counted on five representative fields/well from six replicate wells, (original magnification ×200); (**B**) *Nf1^+/−^* MSPCs had significantly increased adhesion to CH296 coated plates in comparison to WT MSPCs (*** *p* < 0.001 for CH296 coated *Nf1^+/−^* MSPCs *vs.* CH296 coated WT MSPCs); (**C**) Preferential adhesion to fibronectin binding sites, H296 and CH271, was assessed. *Nf1*^+/−^ MSPCs exhibited significantly greater adhesion to CH271 as compared to H296. (*** *p* < 0.001 for CH271 coated *Nf1^+/−^* MSPCs *vs.* CH271 coated WT MSPCs); (**D**) Expression of CH271 receptor, CD49e, was quantified by flow cytometry, demonstrating significantly increased expression of CD49e in *Nf1^+/−^* MSPCs in comparison to WT. The green lines represent isotype controls while the green solid areas represent the experimental samples. Data are one representative result of three independent experiments, and each experiment was performed with different MSPCs culture isolated from individual mice.

*Nf1*^+/−^ MSPCs showed significantly increased adhesion to recombinant fibronectin fragment, CH296, pre-coated plates as compared to WT controls (Figure 3A; *p* < 0.001, Figure 3B). Fibronectin’s binding sites, H296 and CH271, were analyzed for preferential affinity in *Nf1*^+/−^ MSPCs. H296 and CH271 specific adhesion assays demonstrated a significant increase *in Nf1*^+/−^ MSPC adhesion in CH271 assays but not H296 assays as compared to WT (*p* < 0.001, Figure 3C). By contrast, WT MSPCs did not show preference to either CH271 or H296. To further characterize the increased affinity of *Nf1*^+/−^ MSPCs for CH271, expression of its receptor, CD49e (also known as integrin α5 or fibronectin receptor α), was quantified by flow cytometry. The expression level of CD49e in *Nf1*^+/−^ MSPCs was significantly increased as compared to WT MSPCs (Figure 3D). We also analyzed CD49d, the H296 receptor, and found no statistical difference in expression between WT and *Nf1*^+/−^ MSPCs (data not shown).

### 2.4. Hyper Activation of the PI3-K and MAPK Pathways in Nf1^+/−^ MSPCs

**Figure 4:**
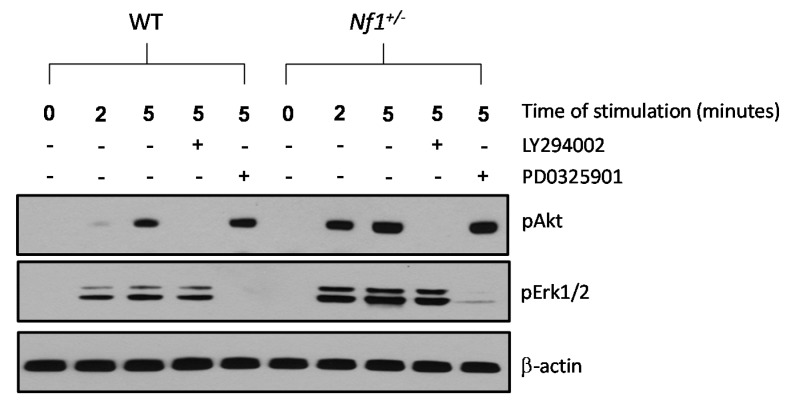
*Nf1^+/−^* MSPCs exhibit increased Akt (also known as protein kinase B) and extracellular-signal-regulated protein kinase (Erk)1/2 phosphorylation, which can be inhibited by LY294002 and PD0325901, respectively. Phosphorylation of Akt and Erk1/2 was determined by Western blot in WT and *Nf1^+/−^* MSPCs following 10% FBS stimulation in the presence or absence of PI3-K inhibitor, LY294002, or MAPK inhibitor, PD0325901. Data represents one of three independent experiments, and each experiment was performed with different MSPCs culture isolated from individual mice.

Serum starved WT and *Nf1^+/−^* MSPCs were stimulated with 10% FBS to assess activation of the PI3-K and MAPK pathways downstream of Ras. Baseline expression of phosphorylated (p) Akt (also known as protein kinase B) and extracellular-signal-regulated protein kinase (Erk) 1/2 in *Nf1^+/−^* and WT MSPCs were undetectable. However, stimulation with 10% FBS for 2 and 5 min showed significantly enhanced expression of pAkt and pErk1/2 in *Nf1^+/−^* MSPCs compared to WT MSPCs. Increased pAkt levels were restored to baseline by 30 min pretreatment of a PI3-K inhibitor, LY294002, in both WT and *Nf1^+/−^* MSPCs. Likewise, pErk1/2 expression was significantly reduced in the presence of a MEK inhibitor, PD0325901, in both WT and *Nf1^+/−^* MSPCs (Figure 4). The pretreatment of LY294002 and PD0325901 has also been prolonged to 4 h, and the results did not show a significant pathway cross talk occurred between these two inhibitors (Figure S2).

Cell viability was not affected by these inhibitors at the concentration applied here. Collectively, these data demonstrated that *Nf1^+/−^* MSPCs exhibit hyperactivation of both the PI3-K and MAPK signaling axes, which can be restored to baseline by pathway specific pharmacologic inhibitors.

### 2.5. Enhanced Nf1^+/−^ MSPCs Migration and Adhesion Is Rescued by LY294002 and PD0325901

**Figure 5:**
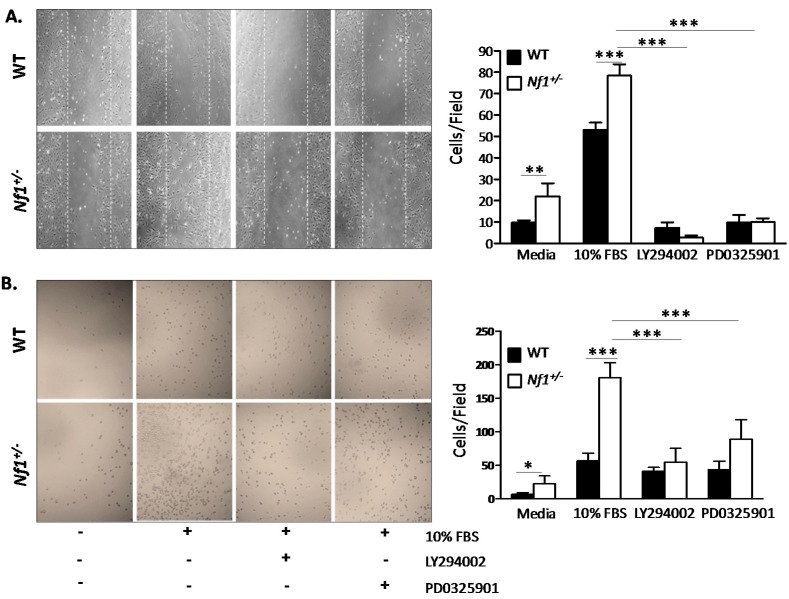
Migration and adhesion of *Nf1^+/−^* MSPCs was mediated by mitogen activated protein kinase (MAPK) and phosphoinositide 3-kinase (PI3-K) pathways. (**A**) Representative high power fields (20× objective lens) of wound healing assays for WT and *Nf1^+/−^* MSPCs cultured with serum free media or 10% FBS in the presence or absence of either LY294002 or PD0325901. *Nf1^+/−^* MSPCs have enhanced migration in comparison to WT in serum free or 10% FBS supplemented media, which was significantly decreased by LY294002 and PD0325901 (** *p* < 0.01 for *Nf1^+/−^* MSPCs *vs.* WT MSPCs cultured in media; *** *p* < 0.001 for 10% FBS treated *Nf1^+/−^* MSPCs *vs.* 10% FBS treated WT MSPCs; *** *p* < 0.001 for LY294002 or PD0325901 treated and untreated *Nf1^+/^^−^* MSPCs in the presence of 10% FBS); (**B**) Representative high power fields (20× objective lens) from CH296 adhesion assays for WT and *Nf1^+/−^* MSPCs performed in serum free or 10% FBS supplemented media in the presence or absence of LY294002 or PD0325901. The adhesion of *Nf1^+/−^* MSPCs was significantly increased in comparison to WT MSPCs in either serum free or 10% FBS supplemented media. Adhesions were significantly reduced in the presence of LY294002 and PD0325901 (* *p* < 0.05 for *Nf1^+/−^* *vs.* WT MSPCs in serum free media; *** *p* < 0.001 for *Nf1^+/−^* *vs.* WT MSPCs in 10% FBS supplemented media; *** *p* < 0.001 for LY294002 or PD0325901 treated and untreated *Nf1^+/^^−^* MSPCs in the presence of 10% FBS). Data are represented as mean ± SD from three individual experiments, and each experiment was performed with different MSPCs culture isolated from individual mice.

In order to determine the functional implications of pharmacologically inhibiting hyperactivated PI3-K and MAPK pathways in *Nf1^+/−^* MSPCs, wound healing and adhesion assays were performed to assess migration and adhesion of MSPCs in the presence of LY294002 or PD0325901 inhibitors. In comparison to WT, *Nf1^+/−^* MSPCs demonstrate increased migration in response to either media alone or media supplemented with 10% FBS, which was significantly attenuated by LY294002 or PD0325901 (*p* < 0.001, Figure 5A). Migration of *Nf1^+/−^* MSPCs *vs.* WT MSPCs responded to different concentrations of PD0325901, as shown in Figure S3. A similar trend was noted in the adhesion assay where *Nf1^+/−^* MSPCs were significantly more adhesive in comparison to WT in response to either media alone or 10% FBS stimulation. Likewise, adhesion was markedly reduced in the presence of LY294002 and PD0325901 (*p* < 0.001, Figure 5B). The efficacy of PI3-K and MAPK inhibitors to significantly attenuate the enhanced signal transduction, migration, and adhesion of MSPCs with *Nf1* haploinsufficiency suggests that these two pathways may play important role in mediating *Nf1^+/−^* MSPC gain-in-functions.

## 3. Discussion

NF1 is a heritable or spontaneous autosomal dominant disease, affecting neural tissues, skin, and skeleton. Skeletal abnormalities such as scoliosis, sphenoid wing dysplasia and osteopenia are common manifestations of NF1. Elevated bone resorption has been proven to contribute to the abnormal bone remodeling in NF1 [24]. During bone resorption, the abundant factors released from the bone matrix results in recruitment and differentiation of MSPCs for the following bone remodeling. In addition, fibroblasts are one of the major cell components of neurofibromas. Through current studies indicated that endoneurial fibroblasts are neural crest derivative [25], whereas perineurial fibroblasts, which are involved in the nerve degeneration and regeneration of NF1, are mesenchymal origin [26,27]. Nevertheless, the relevance between MSPCs and fibroblasts in neurofibromas still needs further investigation. Taken together, MSPCs and its progeny cells may play a critical role in the pathogenesis of NF1.

Just like HSPCs, MSPCs migrate and localize throughout the whole body. Several studies have demonstrated migratory capabilities of MSPCs are mediated through numerous chemokine-receptor interactions and cell adhesion molecules [11,28,29,30,31,32]. Takashima and colleagues have reported the presence of neuroepithelium-derived and neural crest-derived MSPCs in cell populations of femoral and tibial bones in postnatal mice [33]. A study performed by Mansilla *et al.* indicated that peripheral blood collected from a group of 15 acute burn victims showed a larger percentage of MSPCs compared to a group of 15 healthy individuals [34]. Collectively, these studies demonstrated the migratory capabilities of MSPCs and implicated their involvement in the regenerative process *in vivo*. However, little is known about the molecular mechanism(s) underlying MSPC migration under the circumstance of NF1, which may further inform the role of MSPCs in the pathogenesis of NF1 such as abnormal bone remodeling and tissue repair.

Ponte *et al.* [35] have shown that bone marrow MSPCs have the tendency to migrate towards certain growth factors and chemokines when cultured *in vitro*. Using transwell migration assay, platelet derived growth factor (PDGF) was demonstrated to have the most potent MSPC attractive effect. Other studies have shown that when plated with vascular smooth muscle cells, PDGF induces an increase in MAPK activity [36]. As an inflammatory cytokine, tumor necrosis factor-alpha (TNF-α) is known to significantly increase Ras activity [37]. Furthermore, TNF-α mediated stimulation of bone marrow MSPCs was shown to improve their response to certain chemokines such as stromal cell derived factor 1 (SDF-1) and RANTES and enhance migratory functions compared to non-stimulated control [38]. These aforementioned studies implicate a critical role for Ras and its downstream signaling pathway in MSPC migratory functions. We hypothesized that hyperactivation of p21-Ras pivotally underpins the enhanced cellular adhesion and migratory functions of *Nf1^+/−^* MSPCs. *Nf1^+/−^* MSPCs demonstrate hyperactivation of p21 Ras secondary to neurofibromin haploinsufficiency. The MAPK and PI3-K pathways are direct downstream effectors of p21-Ras. In our study, hyperactivity of the PI3-K and MAPK pathways in *Nf1^+/−^* MSPCs was confirmed by Western blot demonstrating increased levels of pAkt and pErk1/2. Through the adhesion and wound healing assays, we have shown that *Nf1^+/−^* MSPCs exhibit significantly enhanced adhesion and migration capabilities compared to WT MSPCs. Furthermore, inhibition of MAPK and PI3-K pathways in *Nf1^+/−^* MSPCs using PD0325901 or LY294002, respectively, resulted in a dramatic decrease in the enhanced migratory and adhesive capacity of *Nf1^+/−^* MSPCs to levels comparable to WT cells. Similarly, transwell migration assay also showed the enhanced migration in *Nf1^+/−^* MSPCs was restored to the same level as WT MSPCs in the presence of PD0325901 or LY294002 (Figure S4). However, Koivunen *et al*. [39] demonstrated that neither the function of wound healing nor the Ras-MAPK activity were enhanced in epidermal cells of NF1 patients, indicated that NF1 gene play a key regulator of Ras signaling only in some kinds of cell types.

Studies have also demonstrated that Ras signaling regulated Nf1 heterozygous cell migration in other cell types, indicating that the defects of cell motility may be a common characteristic of neurofibromin deficiency. Neurofibromin-deficient macrophages demonstrated increased proliferation, migration, and adhesion, which were regulated by Ras-MAPK signaling [40]. Similarly, osteoclasts derived from NF1 patients demonstrated increased migration and adhesion capacity associated with hyperactivity of MAPK pathways [41]. While in *Nf1^−/−^* Schwann cells, not only MAPK but also PI3-K pathways contributed to the increased migration capacity [42], which was consistent with our results in this study, implying synergistic effect of these two pathways in the migratory phenotype in certain cell types. In addition to the impaired osteoblastic differentiation reported by us previously, the enhanced migration and adhesion in MSPCs may also be associated with the osseous manifestations in NF1 patients.

## 4. Experimental Section

### 4.1. Animals and Materials

*Nf1* mice were obtained from Dr. Tyler Jacks at the Massachusetts Institute of Technology (Cambridge, MA, USA) in a C57BL/6J.129 background and backcrossed for 13 generations into a C57BL/6J strain. *Nf1^+/−^* mice were genotyped by polymerase chain reaction (PCR) as previously described [22]. These studies were conducted with a protocol approved by the Indiana University Laboratory Animal Research Center using four to eight week old WT and *Nf1^+/−^* mice.

### 4.2. Isolation and Expansion of MSPCs

MSPCs were generated from WT and *Nf1^+/−^* mice as previously described [43]. Briefly, four to eight week old WT and *Nf1^+/−^* mice were sacrificed by CO_2_ inhalation followed by cervical dislocation. Bone marrow cells were collected by flushing the femurs and tibias with Isccove’s Modified Dulbecco’s Medium (IMDM, Gibco-invitrogen, Carlsbad, CA, USA) containing 2% FBS using a 23-gauge needle. Bone marrow mononuclear cells (BMMNCs) were then separated by low-density gradient centrifugation using Ficoll Hypaque. Cells were washed twice with IMDM and suspended in mouse MesenCult basal medium containing mesenchymal cell stimulating supplements (Stem Cell Technologies Inc., Vancouver, BC, Canada). The cells were then plated at a density of 2 × 10^7^ cells in a 10-cm tissue culture dish (BD Falcon, Franklin Lakes, NJ, USA).

### 4.3. Phenotypic Analysis of MSPCs

The expression of cell surface markers were analyzed to measure the purity of the cultured MSPCs. One hundred thousand cells were re-suspended in 100 µL of 0.1% BSA/PBS and stained with FITC-anti-mouse CD44, R-phycoerythrin (R-PE)-conjugated anti-mouse CD49e, R-PE-conjugated anti-mouse CD29, purified rat anti-mouse CD105, anti-rat R-PE antibody, c-Kit, CD34, CD13, Mac-1, B220, and Gr-1 antibodies (BD Pharmingen, San Diego, CA, USA) for 30 min at 4 °C. After washing with PBS twice, the labeled cells were analyzed via flow cytometery (Becton-Dickinson, San Jose, CA, USA).

### 4.4. Cellular Morphology

Morphological analysis was determined by plating 1 × 10^4^ cells/well in a plastic 8-well chamber slide (Ibidi, Martinsried, Germany) for 24 h in MesenCult media with supplemental added. Cells were then fixed with 3.7% formaldehyde and permeabilized with 0.1% Triton X/PBS for 5 min and blocked for 1 h in 5% Milk/PBS before staining with DAPI and 400nM FITC-phalloidin (Sigma P5282, St. Louis, MO, USA). Cells were examined and images were acquired using deconvolution microscope (Delta vision Elite, Applied Precision, Mississauga, ON Canada). Average ratio of nuclear to cytoplasmic areawas calculated using ImageJ software (NIH, Cambridge, MA, USA).

### 4.5. Actin Polymerization

Actin polymerization was measured as described previously [38]. Briefly, Cells were starved for 2 h and then treated with or without 10% FBS for 30 s, 1 min and 5 min and fixed with 3.7% formaldehyde in PBS and stained with 400 nM FITC phalloidin (Sigma P5282). Cells were then incubated for 10 min at 37 °C and transferred to 4 °C for overnight staining. The following day, cells were washed and analyzed by flow cytometer (Becton-Dickinson).

### 4.6. Adhesion Assay

MSPCs were starved in DMEM for 2 h, detached with trypsin, washed, and resuspended in serum-free media with 0.01% BSA, and then plated in pre-coated plates containing 8 µg/mL CH296, 30 µg/mL CH271 or 20 µg/mL H296 (Takara Bio., Otsu, Japan). The cells were allowed to attach and spread for 1 h at 37 °C, and then unbound cells were removed by gentle washing with PBS. Attached cells were fixed with 10% formaldehyde, stained with 0.1% crystal violet and counted [44].

### 4.7. Wound Healing Assay

The migratory ability of MSPCs was measured using a wound-healing assay [45]. In brief, 1 × 10^5^ cells were plated on BD Falcon 12-well tissue culture plates at approximately 70% confluence. A wound was then created by scratching a linear ridge in the cell monolayer using a 1000 µL pipette tip (approximately 1.3 mm wide), ensuring that all wounds were of equivalent width. To prevent width change secondary to cellular proliferation, MSPC cultures were treated with 10 µg/mL of mitomycin C (Sigma M7949) 1 h prior to wound formation to block cellular mitosis. In experiments with inhibitors, LY294002 (10 μM) or PD0325901 (100 nM), cells were co-incubated for 1 h with mitomycin C. Upon wound generation, cell culture media was replaced with fresh media, and wound closure was allowed to proceed for 24 h prior to Hema-3 staining (Fisher Scientific Company LLC, Kalamazoo, MI, USA).

### 4.8. Western Blot

WT and *Nf1^+/−^* MSPCs were deprived of serum and growth factors for 24 h. For the stimulation only group, cells were stimulated with 10% FBS for 2 or 5 min. For the inhibitor group, cells were pre-incubated with LY294002 (10 μM) or PD0325901 (100 nM) for 30 min, followed by stimulation with 10% FBS for 5 min. Cells were lysed in nonionic lysis buffer (20 mM Tris-Cl, 137 mM NaCl, 1 mM EGTA (ethylene-glycol-tetra-acetic acid), 1% Triton X-100, 10% glycerol, 1.5 mM MgCl_2_) containing protease and phosphatase inhibitors (Amersham Pharmacia Biotech, Piscataway, NJ, USA). The protein concentration of the lysates was normalized using a bicinchoninic acid (BCA) assay kit (Pierce, Rockford, IL, USA). Proteins were separated by 12.5% sodium dodecyl sulfate polyacrylamide gel electrophoresis (SDS-PAGE) and transferred to high-quality polyvinylidene difluoride membranes (Roche Diagnostics, Indianapolis, IN, USA) in a Tris (20 mM), glycine (150 mM) and methanol (20%) buffer at 250 mV for 2 h. After blocking in 5% nonfat dry milk in phosphate buffered saline tween-20 (PBST), the membranes were incubated with primary antibodies at 1:1000 dilution (phospho-Erk, phospho-Akt, β-Actin; Cell Signaling, Danvers, MA, USA) in 5% milk in PBST overnight at 4 °C. Following overnight exposure, the membranes were washed three times with PBST and incubated with secondary antibodies (anti-rabbit, anti-mouse, GE Healthcare UK Limited, UK) conjugated with horseradish peroxidase at 1:5000 dilution in 5% milk in PBST for 1 h at room temperature. Membranes were washed again in PBST three times at room temperature. Membranes were exposed to SuperSignal West Pico Chemiluminescent Substrate (Thermo Scientific, Rockford, IL, USA) and protein bands were visualized on an X-ray film (GeneMate, Kaysville, UT, USA).

### 4.9. Statistical Analysis

All statistical analyses were performed with GraphPad Prism 5.0. Unpaired two-tailed student’s *t* tests were used for two variable comparisons. Two-way ANOVA with Bonferroni *post-hoc* corrections was performed for experiments with two different categorical independent variables. Data are presented as mean ± SD. The number of biologically-independent replicates, and significance levels were shown in the figure legends. Differences were considered statistically significant at *p* < 0.05.

## 5. Conclusions

In sum, these data indicated a critical role for *Nf1* in regulating multiple MSPCs functions, including migration and adhesion through both the PI3-K and MAPK pathways. Inhibition of these hyperactive pathways independently in *Nf1^+/−^* MSPCs resulted in attenuation of their enhanced propensity to migrate and adhere. Further characterization of the role of PI3-K and MAPK signal transduction in MSPC function is of critical importance to providing continued insight into the homing and migratory capabilities of MSPCs with potential future therapeutic applications.

## Supplementary Materials

Supplementary materials can be found at http://www.mdpi.com/1422-0067/16/06/12345/s1.
