# Supplementary material for: Hyperactive RAS/PI3-K/MAPK Signaling Cascade in Migration and Adhesion of Nf1 Haploinsufficient Mesenchymal Stem/Progenitor Cells
Source: Int J Mol Sci. 2015 Jun 1;16(6):12345–59. doi: 10.3390/ijms160612345 (PMC4490447; doi:10.3390/ijms160612345)
Supplement: Supplementary file 1 [file ijms-16-12345-s001.pdf]

Supplementary Information

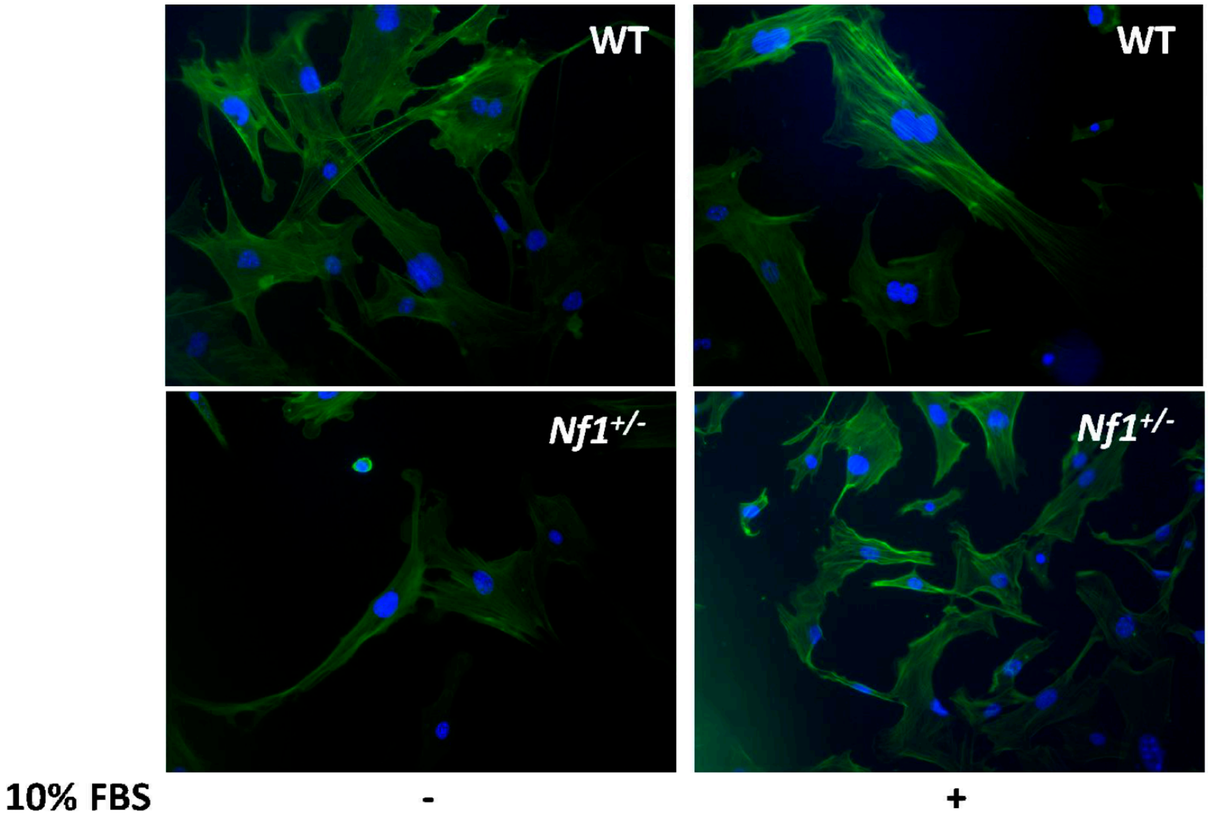

Figure S1. Increased F-actin content in *Nf1*$^{+/-}$MSPCs compared with WT MSPCs. Cells were starved for 2 h followed by treatment with 10% FBS for 30 s. Morphology of WT and *Nf1*$^{+/-}$MSPCs were imaged by confocal microscopy (original magnification ×400). Cells were stained with 400nM FITC-phalloidin and DAPI.

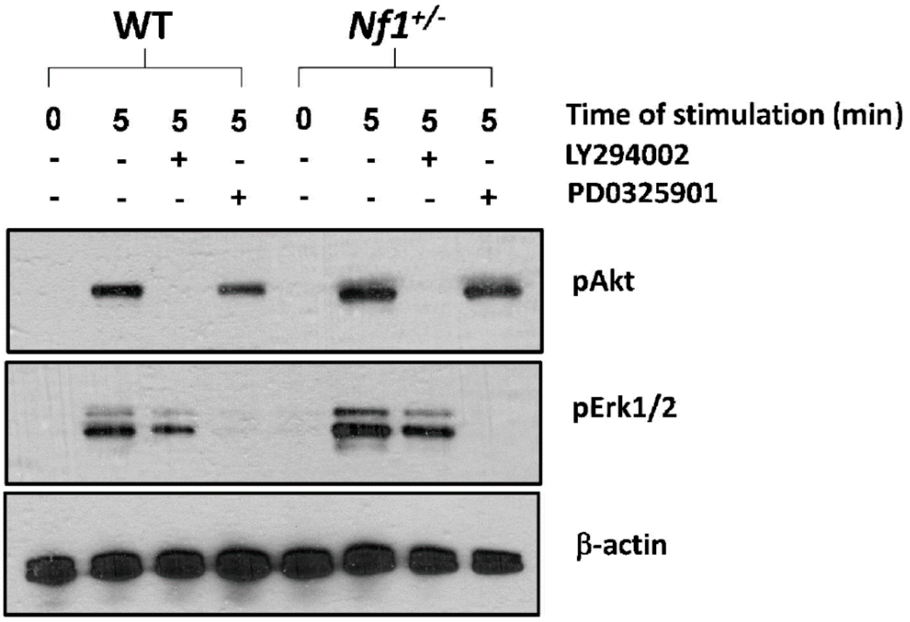

Figure S2. Prolonged pretreatment of LY294002 or PD0325901 in *Nf1*$^{+/-}$and WTMSPCs did not show a significant pathway cross talk. Cells were pretreated with LY294002 or PD0325901 for four hours, followed by 10% FBS stimulation for 5 min. Data represents one of three independent results.

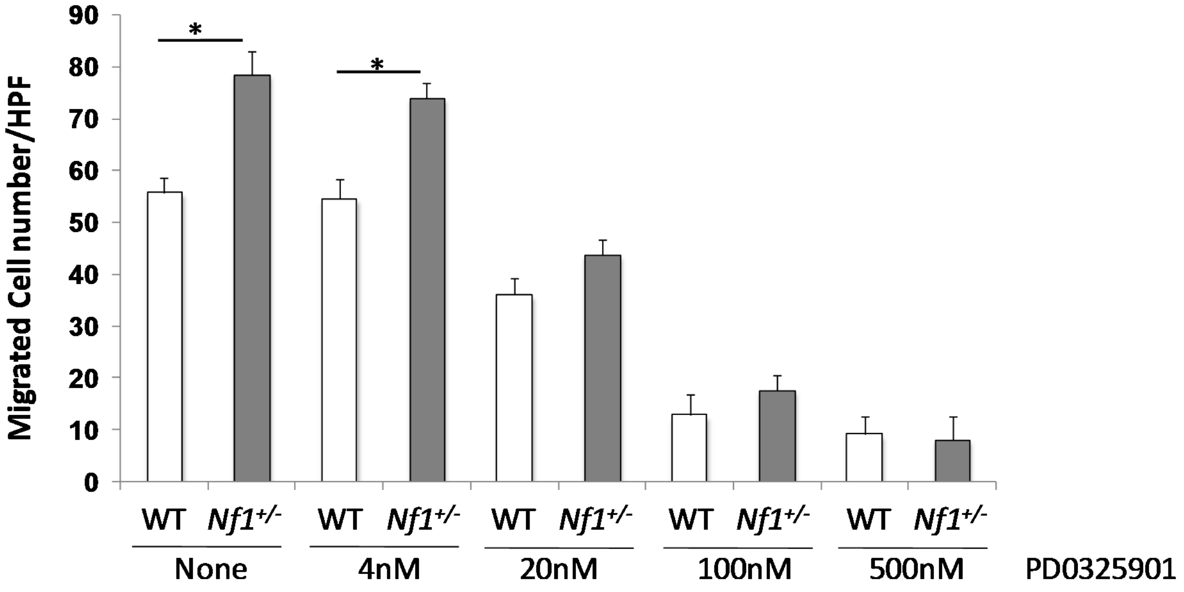

A

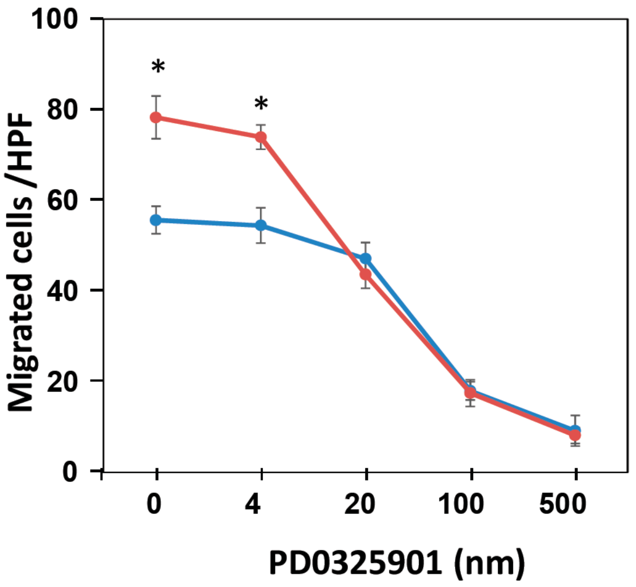

B

Figure S3. Migration of *Nf1*$^{+/-}$MSPCs *vs.* WT MSPCs at different concentrations of PD0325901. Wound healing assays for WT and *Nf1*$^{+/-}$MSPCs were performed with 10% FBS in the presence of different concentrations PD0325901. (**A**) *Nf1*$^{+/-}$MSPCs have enhanced migration in comparison to WT MSPCs in the absence or 4 nM of PD0325901 (* $p < 0.05$), which was significantly decreased by higher concentration of this inhibitor; (**B**) Dose response curve of migrated *Nf1*$^{+/-}$MSPCs (red line) or WT MSPCs (blue line) after PD0325901 treatment.

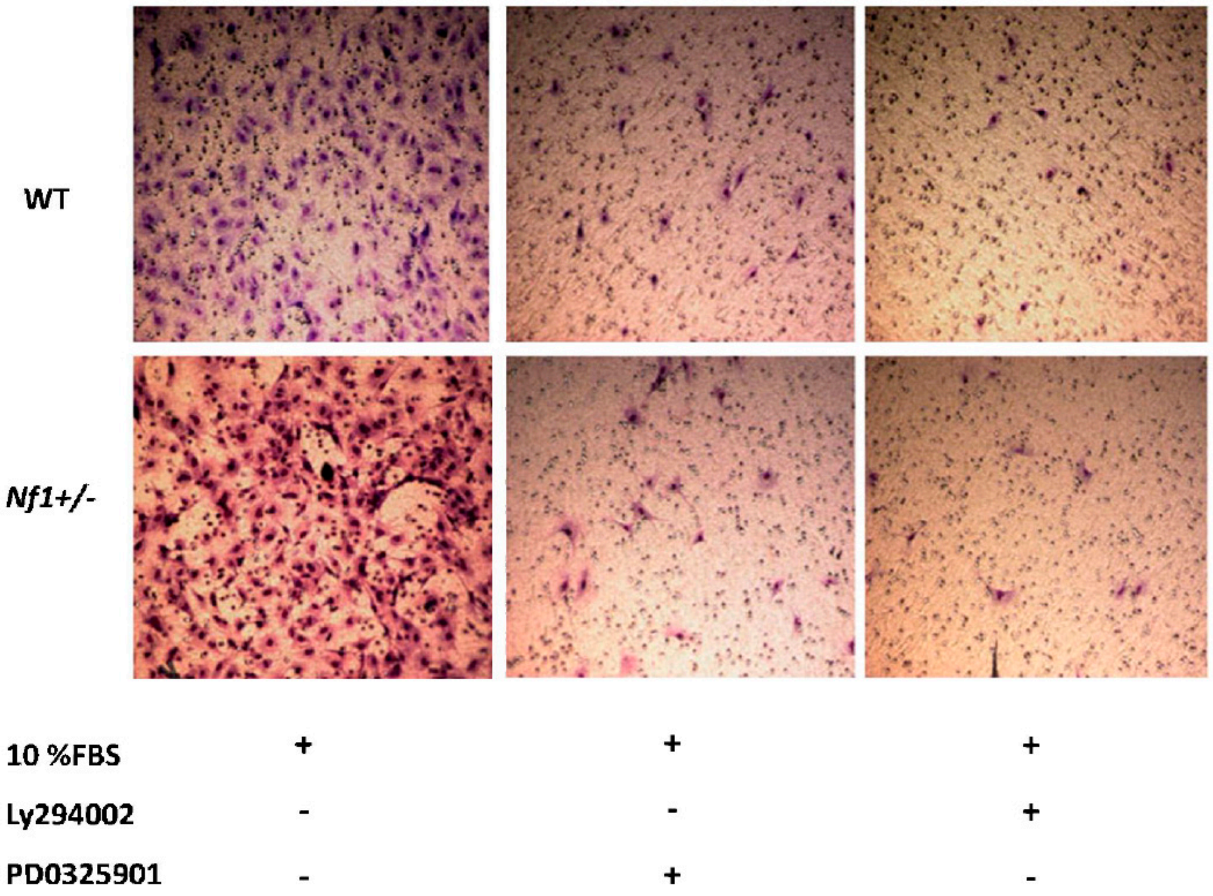

Figure S4. Transwell assays for WT and *Nf1*$^{+/-}$MSPCs were performed in 10% FBS supplemented media in the presence or absence of LY294002 or PD0325901. Cells grown in MesenCult medium were harvested and resuspended in DMEM with 10% FBS. Eight µm porous polycarbonate membrane Transwell was prepared by coating the bottom of the membrane with 8 µg/mL fibronectin for 2 h under 37 °C. The membrane was washed with PBS, and 500 µL of DMEM with 10% FBS was added into the bottom well while the top well received 100 µL of 1 $\times 10^5$cells/mL DMEM and 10% FBS. The plate was placed under 37°C incubation for 24 h before being stained with Hema-3 staining kit and analyzed (original magnification ×200).
